# Integrating High-Throughput Sequencing Data from Herbarium and Contemporary Samples Reveals a Novel Carlavirus Long Established in European Beech

**DOI:** 10.3390/microorganisms14061340

**Published:** 2026-06-15

**Authors:** Pier P. M. de Koning, Anne K. J. Giesbers, Susanne von Bargen, Stephanie T. G. Rensen, Carmen Büttner, Marcel Westenberg, Marleen Botermans, Artemis Rumbou

**Affiliations:** 1 Netherlands Institute for Vectors, Invasive Plants and Plant Health (NIVIP), National Plant Protection Organization (NPPO), Netherlands Food and Consumer Product Safety Authority (NVWA), Geertjesweg 15, 6706 EA Wageningen, The Netherlands; 2 Division of Plant Pathology and Plant Protection, Albrecht Daniel Thaer-Institute of Agricultural and Horticultural Sciences, Faculty of Life Sciences, Humboldt-University of Berlin, Lentzeallee 55/57, 14195 Berlin, Germany

**Keywords:** beech, *Fagus sylvatica*, carlavirus, HTS, data sharing, virome, baseline studies

## Abstract

High-throughput sequencing (HTS) was applied to investigate the virome of European beech (*Fagus sylvatica*) from asymptomatic leaves and symptomatic leaves exhibiting chlorosis, line patterns and malformation. Total RNA extracted from six samples, including herbarium material collected in 1967 and 1968 and contemporary samples from France, Germany, and The Netherlands, was subjected to Illumina sequencing followed by de novo assembly, sequence similarity searches and phylogenetic analyses. In each sample, contigs belonging to a previously undescribed virus within the genus *Carlavirus* were obtained. The virus was tentatively named beech carlavirus. No additional virus contigs were detected in the samples. The detection of the virus over more than five decades and in three European countries indicates its long-term and a probable wider occurrence and circulation. Moreover, its prolonged unnoticed presence suggests that it does not induce noticeable and acute disease outbreaks. These findings underscore the value of integrating historical and recent field samples through collaborative data sharing to improve insight into virus diversity and ecology in forest trees.

## 1. Introduction

Historically, forest trees have received little attention in plant virus research, possibly because they were difficult to investigate using classical diagnostic methods. European beech (*Fagus sylvatica*, also known as common beech) is an example of a tree species whose virome remains largely unexplored. Early reports of viral infections in beech in Europe emerged in the 1960s [1]. This initiated systematic investigations into virus–host interactions in beech. Nienhaus and Castello [2] summarized observations of virus-like symptoms in broadleaf forest trees. In degenerating beech trees at different forest sites of West Germany, several virus species were detected, among which bean yellow mosaic virus (BYMV; *Potyvirus phaseoluteum*), brome mosaic virus (BMV; *Bromovirus BMV*) and cherry leaf roll virus (CLRV; *Nepovirus avii*) [3,4] were the most commonly reported [5,6,7]. These viruses were initially identified by electron microscopy and a serological test and several were further characterized. However, the causal agent of the reported leaf symptoms and degeneration was not proven. Collectively, these foundational studies represented the first viral infections in European beech and laid the groundwork for contemporary research. No other viruses have been described from European beech in the decades following these initial discoveries [8,9,10,11].

Over the past two decades, high-throughput sequencing (HTS) has become widely adopted in plant virology as a powerful method to detect and/or identify plant viruses, including novel viruses, without requiring prior knowledge. HTS has, for instance, been applied to sequence historical virus collections (e.g., herbarium material), enabling the recovery and characterization of viral genomes that were previously undetectable using classical diagnostic methods and for which no sequence data were available [12,13]. Moreover, HTS is widely used in virus baseline studies to characterize the virome in both symptomatic and asymptomatic plants, including studies focused specifically on forest tree species [14].

Combining sequence data from historical material (e.g., herbarium specimens) with contemporary field samples from baseline studies provides insights into the geographical distribution of viruses, documents their long-term presence, refines potential associations between infection and symptom expression, and strengthens pest risk assessments as well as evidence-based regulatory decision-making [15]. Recent publications have highlighted the need for baseline studies and recommend systematic investigations of virus diversity and distribution across countries, hosts and ecosystems [16,17]. In response, several initiatives have been launched to perform such baseline studies [18,19,20,21].

Although research on viruses in forest and urban trees intensified over subsequent decades, knowledge of virus occurrence remains particularly limited compared with fruit trees [8,9]. To address this gap, the Euphresco project VIRNOTree was launched in 2023 with the aim of characterizing the virome of forest trees. The present study does not represent a systematic investigation but integrates several independent findings from a pre-publication data sharing initiative in which putative novel virus sequences were exchanged among plant virologists. The samples analyzed were originally collected for different purposes: diagnostics, characterization of a historical collection and a limited baseline study in trees.

Within this context, we report the discovery of a putative novel carlavirus (family *Betaflexiviridae*, order *Tymovirales*) infecting European beech. The virus appears to be geographically widespread, having been detected in France, Germany and The Netherlands, and its presence can be traced back to 1967.

## 2. Materials and Methods

### 2.1. Sampling Information

Six European beech leaf samples from France, Germany and The Netherlands were included in this study, because a putative novel carlavirus was detected (Table 1). Three field samples (11647, 11648, 40757) were collected based on the presence of virus-like symptoms from Germany and France. Two samples were obtained from herbarium specimens preserved in the virus symptom herbarium collection at NIVIP, The Netherlands. One additional sample (6165949) did not exhibit virus-like symptoms and was collected in The Netherlands within the framework of the Euphresco VIRNOTree project.

### 2.2. RNA Extraction, Library Preparation and Sequencing

#### 2.2.1. Samples 1 to 3

Samples were sequenced using Illumina technology as described in Rumbou et al. [22]. Total RNA was isolated from an amount of 0.5–1 g of leaf tissue using the InviTrap^®^ Spin Plant RNA Mini Kit (STRATEC Molecular, Berlin, Germany), followed by removal of remaining DNA with rDNase according to the supplier protocol (Macherey-Nagel, Düren, Germany) and RNA purification using NucleoSpin^®^ RNA Clean-up (Macherey-Nagel, Germany). Ribosomal RNA depletion was performed using the RiboMinus Plant Kit for RNA-Seq (ThermoScientific, Darmstadt, Germany). One to two micrograms of RiboMinus RNA of each sample was used for cDNA synthesis with the Maxima H Minus double-stranded cDNA synthesis Kit (ThermoScientific, Darmstadt, Germany) primed with random hexamers. Between 1 and 2 µg of purified double-stranded cDNA from each sample was submitted to BaseClear (Leiden, The Netherlands) for RNA-seq analysis. Libraries were sequenced on a HiSeq 2500 platform (Illumina, San Diego, CA, USA), generating 100 nt paired-end reads, yielding approximately 50–100 Mb of data per sample.

#### 2.2.2. Samples 4 to 6

Samples were sequenced using Illumina technology as described in EPPO [23]. Total RNA was extracted from approximately 1g of leaf tissue using the RNeasy Plant Mini Kit (Qiagen, Venlo, The Netherlands). Library preparation and sequencing were performed at GenomeScan (Leiden, The Netherlands). Ribosomal RNA was depleted with the QIAseq FastSelect rRNA Plant Kit (Qiagen, Venlo, The Netherlands), after which sequencing libraries were prepared using the NEBNext Ultra II Directional RNA library Prep Kit (New England Biolabs, Ipswich, MA, USA). Libraries were sequenced on a NovaSeq 6000 platform (Illumina, San Diego, CA, USA), generating 150 nt paired-end reads and at least 2 Gb data per sample.

### 2.3. HTS Pipeline and Sequence Analysis

Raw sequencing reads were imported into CLC Genomics workbench v 25.0.1 (Qiagen) and processed using the standard workflow [23]. Reads were quality (quality limit: 0.05 and maximum ambiguities: 2) and adapter (using the automatic adapter trimming function) trimmed and de novo assembled. Contigs were retained for downstream analyses when they exceeded 100 nt with minimum read depth of 10. Consensus sequences were screened against locally installed NCBI databases using MegaBLAST (nt) and DIAMOND [24] (nr). Taxonomic visualization of BLAST outputs was carried out with Krona v.2.7.1 using a bitscore threshold of 25 [25]. Putative carlavirus contigs were queried on BLASTn against the NCBI core_nt database on 4 February 2026 to confirm their novelty. Open reading frames (ORFs) were then predicted in Geneious Prime (v 2025.1.2) and translated protein sequences were assessed by BLASTp against the NCBI nr_cluster_seq database.

### 2.4. Phylogenetic Analysis

A reference dataset of carlavirus sequences was compiled from NCBI RefSeq, the ICTV Virus Metadata Resource (VMR_MSL40.v2.20251013) and closely related taxa identified from the BLASTn output (see Section 2.3). Redundant entries, (e.g., duplicates present in both VMR and RefSeq) were removed. The complete list of reference sequences is provided in Table S1. Reference sequences and the putative novel beech carlavirus sequences were aligned using MAFFT v7.490 [26] with default parameters implemented in Geneious Prime v 2025.1.2. The coat protein (CP) and replicase polyprotein ORFs were translated into amino-acid sequences and aligned separately using MAFFT with default settings. Maximum-likelihood phylogenetic trees were inferred from (I) (near-)complete genomic nucleotide sequences and (II) the amino-acid alignments of the CP and replicase polyprotein amino-acid sequences with IQ-Tree v2.3.6 [27], using the substitution models, as selected by ModelFinder [28], GTR+F+I+G4 for the genomic nucleotide dataset and the Q.pfam+F+I+G4 substitution model for both protein datasets. The RefSeq sequence of cherry virus B (*Foveavirus betavii*, NC_076603, family: *Betaflexiviridae*) was used as the outgroup. Branch support was determined using the ultrafast bootstrap (UFboot) with 10,000 replicates [29]. The phylogenetic trees were refined using TreeViewer v2.2.0 [30] by polytomizing branches with a support values below 70% and collapsing distinct clades.

### 2.5. Datamining Using Serratus and NCBI SRA and TSA

To assess whether the putative novel carlavirus was present in publicly available sequencing data, we screened datasets in Serratus and the NCBI Sequence Read Archive (SRA). For Serratus, the “palmID: Viral-RdRP Analysis” (https://serratus.io/palmid, accessed on 30 April 2026) tool was queried using all the carlavirus replicase polyprotein amino-acid sequences as the input, applying default parameters. In parallel, the NCBI SRA (https://www.ncbi.nlm.nih.gov/sra, accessed on 29 January 2026) was queried using the following search string to retrieve RNA-seq runs from European beech published from 2021 onward: (Fagus sylvatica [Organism]) AND (RNA-Seq [Strategy]) AND (“2021/01/01” [Publication Date]: “3000” [Publication Date]). This time restriction was applied because Serratus indexing partially overlaps with SRA content and to focus on more recent datasets. The nucleotide sequence of the putative novel carlavirus isolate WAG0452949 was subsequently used as a query for BLAST searches against the retrieved SRA dataset. Finally, using the same nucleotide sequence, the presence of beech carlavirus in the Transcriptome Shotgun Assembly (TSA) database was assessed on 30 April 2026 by selecting the TSA database in BLASTn and limiting by “Viridiplantae (taxid:33090)” or “Viruses (taxid:10239)”.

### 2.6. Development of an RT-PCR Test

To establish a diagnostic test, an RT-PCR was developed using two primer pairs (Table 2). The primer pair Carla-S14-7254F/Carla-S14-7751R amplified a 498 bp fragment spanning the TGB3–CP region, whereas Carla-S14-7618F/Carla-S14-7751R amplified a 130 bp fragment within the CP gene. From 1 µg of total RNA of samples 1, 2 and 3, cDNA was synthesized in a 20 µL reaction using random hexamer primers and Maxima H Minus Reverse Transcriptase according to the manufacturer’s instructions (Thermo Fisher Scientific, Schwerte, Germany). The reaction was incubated at 25 °C for 10 min to allow primer annealing, followed by cDNA synthesis at 50 °C for 30 min and enzyme inactivation at 85 °C for 5 min. Subsequent PCR amplifications were performed in a 25 µL reaction volume containing 1× DreamTaq Buffer (Thermo Fisher Scientific), 0.8 mM dNTP mix, 0.625 U DreamTaq DNA Polymerase, and 0.5 µM each of forward and reverse primer. The cycling conditions were as follows: initial denaturation at 94 °C for 2 min, followed by 35 cycles of denaturation at 94 °C for 30 s, annealing for 30 s at the primer-specific annealing temperature (58 °C for the first primer pair and 60 °C for the second primer pair), and extension at 72 °C for 30 s, with a final extension at 72 °C for 5 min. Amplicons from both primer pairs were subjected to Sanger sequencing (Macrogen Europe, Amsterdam, The Netherlands) to confirm target specificity.

### 2.7. Testing of Two Additional Symptomatic European Beech Tree Samples with RT-PCR

Leaf samples (E58078, E58081) from two European beech trees growing in Göttingen, Germany, and displaying virus-like symptoms were tested by RT-PCR. Total RNA was extracted according to Boom et al. [31] and subjected to RT-PCR with carlavirus-specific primer sets as described in the previous chapter (Table 2).

## 3. Results

**Figure 1:**
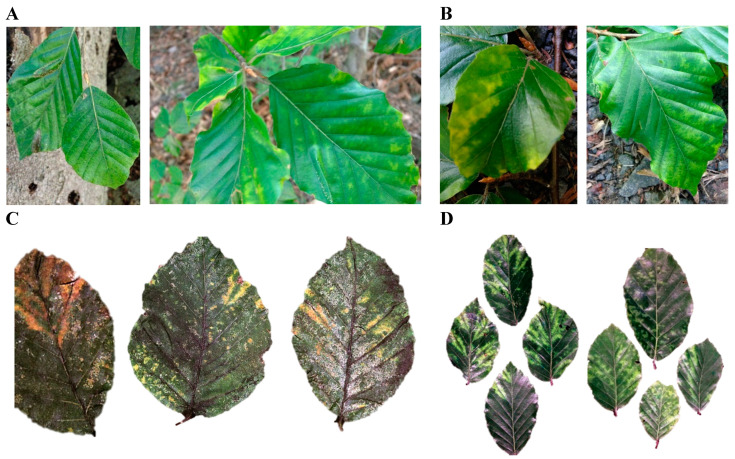
Virus-like symptoms observed on European beech leaves analyzed in this study. (**A**) Sample 11647 (Siebengebirge, Germany; 2014) displaying leaf curling and mottling. (**B**) Sample 11648 (Siebengebirge, Germany; 2014) showing interveinal chlorosis and leaf curling. (**C**) Herbarium specimen WAG0452949 (Groningen, The Netherlands; 1967) and (**D**) herbarium specimen WAG0453175 (Paterswolde, The Netherlands; 1968), both showing V-shaped yellow and orange patterns and chlorotic spots. The images were taken in the 1960s prior to preparing the herbarium specimen.

Five symptomatic leaf samples and one asymptomatic one from European beech were analyzed by HTS (Figure 1 and Table 3). The HTS pipeline detected sequences corresponding to a carlavirus. In five samples, a single viral contig was reconstructed, whereas in sample 2, three distinct contigs were reconstructed, representing different genotypes. In total, eight putative novel carlavirus sequences were obtained, ranging from 8724 to 8872 nucleotides in length. No additional viral contigs were detected. All sequences have been deposited in GenBank under accession numbers PX705376–PX705383, with corresponding SRA BioSamples SAMN53882179–SAMN53882184 and BioProject PRJNA1379522. An overview of sequencing and assembly data is shown in Table 4.

**Figure 2:**
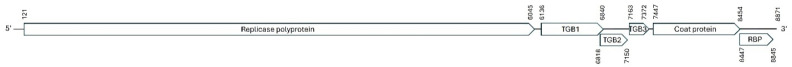
Genome organization of the putative novel carlavirus (isolate WAG0452949). The genome contains six ORFs encoding a replicase polyprotein, the triple gene block proteins (TGB1–TGB3), a coat protein (CP), and a ribonucleoprotein (RBP). Numbers above the genome indicate the start and end nucleotide positions of each ORF.

The putative novel carlavirus nucleotide sequences were queried using BLASTn. The closest match was birch carlavirus (NC_076638.1), sharing 67–69% pairwise nucleotide identity and 69–75% query coverage (Table 5). Genome annotation identified six ORFs typical for members of the genus *Carlavirus* encoding a replicase polyprotein, the triple gene block proteins (TGB1-TGB3), the CP and a ribonucleoprotein (RBP) (Figure 2). BLASTp analyses of the replicase polyprotein and CP yielded the highest similarity to birch carlavirus proteins (YP_010799375.1 and YP_010799379.1) with only 66–67% and 61–63% amino-acid identity, respectively (Table 5). According to the species demarcation criteria for carlaviruses (<80% amino-acid identity in either the replicase polyprotein or the CP), these results indicate that the recovered sequences represent a putative novel carlavirus species [32,33].

To determine whether the eight putative novel carlavirus sequences represent a single species, pairwise comparisons were performed using the genomic nucleotide sequences as well as the deduced amino-acid sequences of the replicase polyprotein and CP. The nucleotide sequences shared 78.8% to 95.3% pairwise identity, while the amino-acid sequences of the replicase polyprotein and CP shared 88.8 to 98.1% and 94.9 to 99.4% pairwise identity, respectively. Based on the established species demarcation criteria, these values indicates that all eight sequences belong to the same virus species.

**Figure 3:**
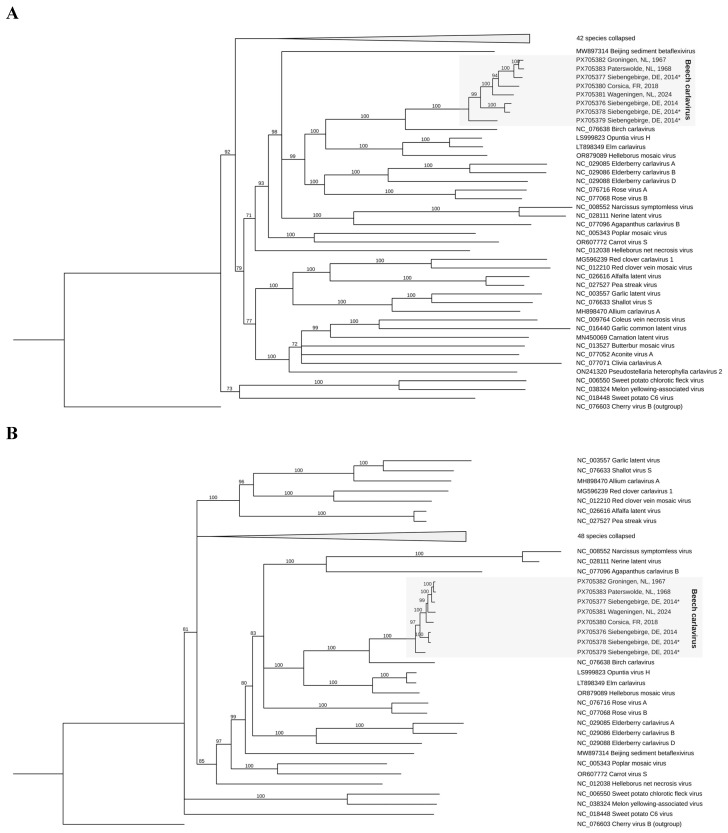

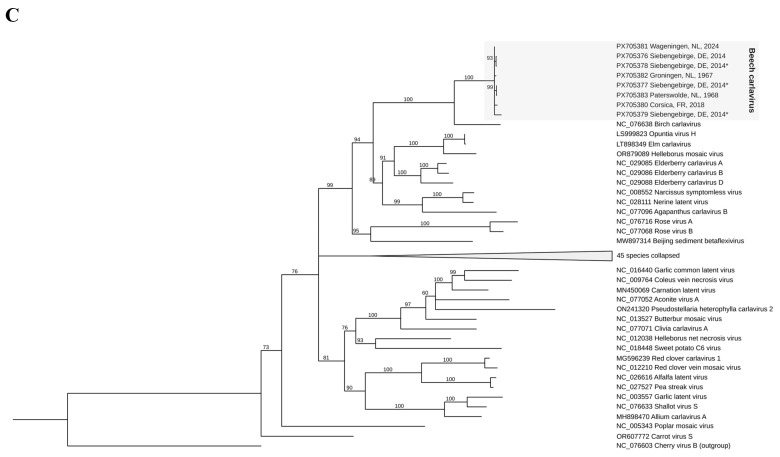
Maximum-likelihood phylogenetic trees including reference carlaviruses species and the putative novel beech carlavirus (shaded in gray). Asterisks (*) denote sequences originating from the same sample. Branch support values (>70%) indicate UFBoot percentages based on 10,000 bootstraps replicates. (**A**) Tree inferred from (near-)complete genomic nucleotide sequences using the GTR+F+I+G4 substitution model. (**B**) Tree inferred from replicase polyprotein and (**C**) from coat protein amino-acid sequences using the Q.pfam+F+I+G4 substitution model.

In addition, three phylogenetic analyses were conducted using the (near-)complete genomic nucleotide sequences and the deduced amino-acid sequences of the replicase polyprotein and the CP of the putative novel carlavirus (Figure 3). In all three analyses, the sequences obtained in this study clustered together as a distinct, monophyletic group with a branch support value of 100%, within the genus *Carlavirus*. Consistent with the pairwise sequence comparisons and species demarcation criteria, these findings support the assignment of the virus to a new carlavirus species, tentatively designated beech carlavirus.

**Figure 4:**
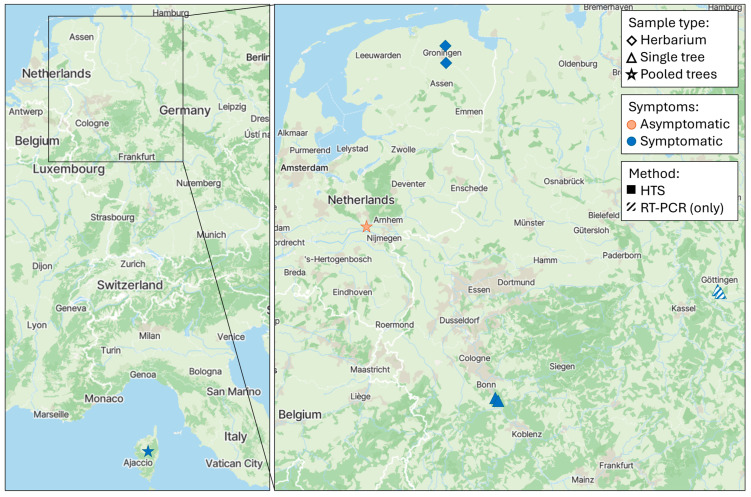
Geographical map displaying the locations of samples that tested positive for beech carlavirus, either by HTS (6 samples) or by RT-PCR only (2 samples).

Beech carlavirus was also detected in samples 1, 2 and 3 by RT-PCR followed by Sanger sequencing (Table 4). Although the RT-PCR was not performed for samples 4 to 6, in silico analysis indicated that the primer binding sites are conserved in these sequences. Furthermore, beech carlavirus was detected in two leaf samples from European beech (E58078, E58081) displaying tip chlorosis and mottling. A geographical map of all the sampling locations of the beech carlavirus positive samples is shown in Figure 4. Finally, data mining in Serratus and NCBI SRA (320 runs across seven BioProjects) and TSA did not result in additional detections of beech carlavirus.

## 4. Discussion

Pre-publication data sharing between two institutes suggested the presence of the same novel carlavirus from beech in several samples, prompting further analysis. Eight putative novel carlavirus genomes were recovered from six European beech leaf samples originating from herbarium and contemporary material. Across three phylogenetic reconstructions, based on (near-)complete genomic nucleotide sequences and the deduced replicase polyprotein and coat protein amino-acid sequences, these isolates consistently grouped into a single, distinct clade within the genus *Carlavirus*. In line with established species demarcation criteria for carlaviruses, and supported by the closest sequence similarity to birch carlavirus, the virus is regarded as a distinct species, tentatively designated as beech carlavirus with the proposed species name *Carlavirus fagi*.

Notably, the eight beech carlavirus genomic sequences displayed substantial sequence diversity, with pairwise nucleotide identities ranging from 78.8% to 95.3%. Such divergence is consistent with patterns reported for other members of the family *Betaflexiviridae*, in which high levels of intraspecies variability are frequently observed [34]. Although revised species demarcation criteria have been proposed for *Betaflexiviridae* [35], these thresholds have not yet been formally adopted by the ICTV which underscores the continued need to interpret classification within a broader phylogenetic and genomic context.

The presence of beech carlavirus in herbarium specimens collected in the late 1960s alongside contemporary field samples indicate that this virus has been circulating in beech populations for at least five decades and is possibly widely distributed in Europe. Yet, it had not been formally described prior to this study. Early studies using electron microscopy, serology and PCR documented several viruses associated with beech; however, none reported carlaviruses [2,3,5,6,7]. Notably, Winter and Nienhaus [3] detected a potyvirus by electron microscopy, from which the particles were morphologically comparable to a carlavirus, but confirmed their finding using a serological test specific for a potyvirus. Therefore, it can be concluded that Winter and Nienhaus [3] described another virus. In the last decade, research on forest tree viromes has increased, but no additional viruses have been formally reported in beech [9]. Vainio et al. [36] included an image of symptomatic beech leaves, resembling those observed in the herbarium samples in this study, suggesting a potential viral etiology and possible infection with beech carlavirus, but did not provide virome data or diagnostic confirmation. To our knowledge, this is the first report of a complete genome from a virus infecting European beech.

Consistent with the absence from the literature, beech carlavirus was not detected in publicly available RNA-sequencing datasets screened via NCBI SRA and TSA and Serratus. This indicates that beech carlavirus sequences have not been previously deposited in public RNA-seq datasets and that the sequences presented here may represent the first publicly deposited sequences of this virus. The absence of detections in NCBI SRA likely reflects the comparatively limited number of European beech RNA-seq runs currently available and the fact that these datasets were generated largely for purposes other than virus discovery; consequently, they may not include tissues, seasons, or symptom states in which high virus titers are expected. Moreover, the lack of detection in Serratus—where SRA datasets from hosts beyond beech were also screened—could be consistent with a restricted host range, but it may equally result from under-sampling of potential hosts. This interpretation aligns with the still limited baseline knowledge of forest tree viromes [36,37]. Overall, these results highlight a surveillance gap and underscore the need for targeted surveys, including systematic HTS-based virome profiling (symptomatic vs. asymptomatic trees, multiple tissues and timepoints) and/or assay-based screening (e.g., RT-PCR), to estimate the prevalence and distribution of beech carlavirus.

The prolonged, previously unrecognized presence of beech carlavirus suggests that it is unlikely to cause acute disease outbreaks and may frequently occur as a low-impact and/or latent infection, with visible symptoms potentially manifesting only occasionally, for instance, when the tree is under stress. This hypothesis is supported by its detection in at least one asymptomatic sample. Nevertheless, beech carlavirus was identified as a sole viral agent detected in five trees exhibiting virus-like symptoms. At present, however, a causal relationship between infection and symptom expression cannot be inferred, especially given the heterogeneity of symptom phenotypes observed across samples.

The symptoms described in this study, particularly those reported from the herbarium samples from The Netherlands showing discolored V-shaped patterns across the secondary veins, can appear superficially similar but should not be confused with beech leaf disease (BLD). This disease is associated with the nematode *Litylenchus crenatae* subspecies *mccanii*, which affects European and American beech (*F. grandifolia*) in North America [38,39]. BLD is typically characterized by banding between the lateral leaf veins, leaf thickening, distortion and leathery texture which markedly differs from the symptoms recorded in this study.

Experience from other forest tree species indicates that variability in symptom expression may reflect complex viromes and mixed infections rather than a single causal agent. For example, individual birch trees have been reported to harbor multiple virus species and different genotypes of the same virus can co-occur within a single host [37]. While this study describes a virus found in single infection in beech, we cannot exclude that additional viruses may occur in beech trees and influence symptom expression. Furthermore, the potential impact of environmental factors such as abiotic stress, co-infection with non-viral pathogens and changing climatic conditions on virus prevalence and symptom development in beech is currently unclear. Taken together, these findings highlight the importance of virus surveillance and the integration of historical and contemporary samples to better understand virus diversity, ecology, and host interactions in forest trees.

The epidemiology of beech carlavirus remains to be determined. At present, there is no scientific evidence for its transmission pathways or dispersal mechanisms (e.g., insect vectors, seed, or pollen). Other carlaviruses are often transmitted vegetatively or via aphids, while seed transmission is uncommon [40]. As transmission via seed for carlaviruses is uncommon, vector-mediated transmission via aphids may be a candidate pathway to investigate further.

## 5. Conclusions

This study demonstrates how HTS, applied to both historical herbarium and contemporary plant material, can substantially advance our understanding of virus–host associations in forest trees. Through pre-publication data sharing, we integrated results that were generated independently at two institutes, providing robust evidence for a novel carlavirus infecting European beech, tentatively named beech carlavirus. The virus appears was detected in samples originating from France, Germany and The Netherlands and has probably a wider distribution in Europe. Its detection over multiple decades, including in asymptomatic trees, is consistent with long-term presence and the potential for latent infections. Virus-like symptoms, including line patterns, were observed in infected beech, but their association with beech carlavirus remains to be formally established, for example, through larger association studies. Based on the current knowledge, we do not have indications that beech carlavirus appears a concern for plant health. Still, its epidemiological impact in natural and managed beech populations, as well as its presence on other continents, remains to be determined.

## Figures and Tables

**Table 1:** Overview of the samples included in this study.

No.	Sample Name	Year	Pool/Single	Sample Type	Country	Location
1	11647	2014	Single	Leaves	Germany	Siebengebirge
2	11648	2014	Single	Leaves	Germany	Siebengebirge
3	40757	2018	Pool (3 trees)	One leaf per tree	France	Corsica
4	6165949	2024	Pool (5 trees)	One leaf per tree	The Netherlands	Wageningen
5	WAG0452949	1967	Single	Herbarium, leaves	The Netherlands	Groningen (city)
6	WAG0453175	1968	Single	Herbarium, leaves	The Netherlands	Paterswolde

**Table 2:** RT-PCR primers used for detection of the novel beech carlavirus.

Set	Name	Gene	Sequence
1	Carla-S14-7254F	TGB3-CP	GCTGGGTATGCTAGTGGACT
	Carla-S14-7751R		GTTTGAGATCATCGACAGGCT
2	Carla-S14-7618F	CP	CAGTGATGAAATGTTGGAGCAG
	Carla-S14-7751R		GTTTGAGATCATCGACAGGCT

**Table 3:** Overview of symptoms observed on the leaves of European beech trees in France, Germany and The Netherlands.

No.	Sample Name	Leaf Symptom Description
1	11647	Curling and mottling
2	11648	Curling and interveinal chlorosis
3	40757	Chlorotic leaf tips and chlorosis along veins
4	6165949	No symptoms observed
5	WAG0452949	V-shaped yellow and orange patterns and chlorotic spots
6	WAG0453175	V-shaped yellow and orange patterns and chlorotic spots

**Table 4:** Overview and HTS metrics of eight putative novel carlavirus contigs identified in European beech.

No.	SRA BioSample	Total Reads per Sample	GenBank Accession	Total Mapped Viral Reads	Mean Read Depth	Sequence Length (nt)	Carlavirus-Specific RT-PCR
1	SAMN53882179	787,486	PX705376	110,642	1547	8774	Positive
2	SAMN53882180	713,488	PX705377	4487	63	8800	Positive
			PX705378	17,283	245	8784	
			PX705379	2567	37	8724	
3	SAMN53882181	6,930,926	PX705380	9579	155	8871	Positive
4	SAMN53882182	16,088,034	PX705381	17,862	298	8872	Not tested
5	SAMN53882183	15,352,090	PX705382	66,216	1010	8871	Not tested
6	SAMN53882184	14,169,406	PX705383	55,277	920	8872	Not tested

**Table 5:** Overview of the NCBI BLASTn/BLASTp results for eight genomic sequences and their corresponding replicase polyprotein and coat protein amino-acid queries; birch carlavirus was the top hit in all cases (E-value of 0.0; NC_076638.1, YP_010799375.1, YP_010799379.1).

No.	GenBank Accession	Genomic or Protein Sequence		Pairwise Identity (%)	Query Coverage (%)
1	PX705376	complete genome	nt	68.06	80
		replicase polyprotein	aa	67.39	100
		coat protein	aa	62.16	96
2	PX705377	complete genome	nt	68.75	76
		replicase polyprotein	aa	66.52	100
		coat protein	aa	61.56	96
	PX705378	complete genome	nt	69.28	74
		replicase polyprotein	aa	67.10	100
		coat protein	aa	62.16	96
	PX705379	complete genome	nt	69.33	69
		replicase polyprotein	aa	66.68	100
		coat protein	aa	62.46	96
3	PX705380	complete genome	nt	68.45	82
		replicase polyprotein	aa	66.73	100
		coat protein	aa	63.30	96
4	PX705381	complete genome	nt	67.33	82
		replicase polyprotein	aa	66.42	100
		coat protein	aa	62.46	96
5	PX705382	complete genome	nt	68.61	75
		replicase polyprotein	aa	66.68	100
		coat protein	aa	61.86	96
6	PX705383	complete genome	nt	68.71	75
		replicase polyprotein	aa	66.60	100
		coat protein	aa	61.56	96

nt: nucleotide; aa: amino acid.

## Data Availability

The nucleotide sequences generated in this study have been deposited in NCBI GenBank (https://www.ncbi.nlm.nih.gov/genbank/) under accessions PX705376-PX705383 and the SRA data in NCBI SRA (https://www.ncbi.nlm.nih.gov/sra) under BioSamples SAMN53882179-SAMN53882184 and BioProject PRJNA1379522.

## Supplementary Materials

The following supporting information can be downloaded at https://www.mdpi.com/article/10.3390/microorganisms14061340/s1, Table S1: Reference sequences for phylogenetic analysis.
